# Downregulation of *Hlx* Closely Related to the Decreased Expressions of *T-bet* and *Runx3* in Patients with Gastric Cancer May Be Associated with a Pathological Event Leading to the Imbalance of Th1/Th2

**DOI:** 10.1155/2012/949821

**Published:** 2012-11-19

**Authors:** Yan Xu, Jingjing Gao, Zhaoliang Su, Xiaoli Dai, Yazhen Li, Yingzhao Liu, Jianguo Chen, Jia Tong, Yun Zhang, Chaoyang Wu, Dong Zheng, Shengjun Wang, Huaxi Xu

**Affiliations:** ^1^Department of Immunology, Institute of Laboratory Medicine, Jiangsu University, Zhenjiang 212013, China; ^2^Department of Laboratory Medicine, The Affiliated People's Hospital, Jiangsu University, Zhenjiang 212001, China

## Abstract

*T-bet* plays an important role in immunoregulation; it induces the differentiation of Th1 together with the homeobox transcription factor gene *Hlx*. Recent studies show that *T-bet* and Th1-associated factors are critical in regulating tumor development. However, the contributions of *Hlx* in the occurrence and development of cancer remain unknown. In this study, the *Hlx*, *T-bet*, *Runx3*, and IFN-**γ** were measured in PBMC from patients with gastric cancer and the correlation between *Hlx* and *T-bet* or IFN-**γ** was assessed. The expression levels of *Hlx*, *T-bet*, and *IFN*-**γ**were significantly decreased, and there was a positive correlation between *Hlx* and *T-bet* or IFN-**γ**. In addition, the *Runx3* expression was also downregulated with the lower *T-bet* mRNA level. These results suggested that the decreased *Hlx* expression was closely associated with *T-bet* and *Runx3* downregulations and may contribute to the development of gastric cancer.

## 1. Introduction

Transcription factors act in concert to induce lineage commitment towards Th1, Th2, Th17, or T regulatory (Treg) cells, and their counter-regulatory mechanisms are shown to be critical for polarization between Th1 and Th2 phenotypes [1–3]. Differentiation of naive T lymphocytes into Th1 cell requires IFN-*γ* (interferon-gamma) which induces the Th1 transcription factor, *T-bet*. But IFN-*γ* just induces initial *T-bet* expression, late expression of *T-bet *to be needs accompanied by the upregulation of the transcription factors *Runx3* and *Hlx *(also known as *Hlx1*, H2.0-like homeobox or *HB24*), and is required to imprint the Th1 cell for IFN-*γ* reexpression [4]. As a strong candidate for a tumor suppressor, *Runx3*, a member of runt-related transcription factors (*Runx1*, *Runx2*, and *Runx3*), has been widely studied in a variety of human cancers [5]. Loss of *Runx3* leading to elevated oncogenic activity was found to be a key event in inducing a precancerous state of the stomach.

The increase in the level of active *p53* protein leads to an inhibition of entry into S-phase or the induction of apoptosis [6]. Thus, the loss or inactivation of *p53* results in the loss of cell-cycle arrest or apoptosis after DNA damage or physiologic stresses. This loss, seen in many human cancers, has been proposed to lead to increased genetic instability, increased accumulation of mutations, and ultimately oncogenesis. Interestingly, a number of studies indicate that tumor-derived mutant forms of *p53*, which are highly expressed in many cancers while losing many of their DNA-damage checkpoint functions function as active transforming genes [7]. These mutant* p53* genes serve as oncogenes that contribute to tumorigenesis [8].

In our previous study, we found that there was a predominant Th2 phenotype in patients with gastric cancer, and we noted that the greatest downregulation of the gene encoding IFN-*γ* was associated with *T-bet* mutation [9, 10]. The *Hlx* is a member of the *Drosophila* homeobox gene *H2.0 *[11, 12]; we selected this gene for study in patients with gastric cancer because of its function in *T-bet* regulation and critical role of *T-bet* in regulating tumor growth. In addition, the expression of *T-bet*, usually accompanied by the upregulation of the transcription factor *Hlx*, was required to imprint the Th cell for interferon-gamma reexpression. However, the contributions of *Hlx* in the occurrence and development of cancer is poorly understood. In the present study, we investigated the correlation between *Hlx* and *T-bet* or IFN-*γ* as well as the expression level of *Runx3* in patients with gastric cancer in order to determine whether *Hlx* contribute to the pathological event leading to the imbalance of Th1/Th2 in gastric cancer.

## 2. Patients and Methods

### 2.1. Patients

90 patients diagnosed newly with gastric cancer treated at the Affiliated People's Hospital of Jiangsu University were included in the study of these patients, 35 females and 55 males, ranging in age from 27 to 65 years. All the patients were untreated for their condition at the time of blood collection. The diagnosis of gastric cancer was based on commonly accepted clinical and laboratory criteria and had been histologically proven, which included 12 papillary adenocarcinoma, 33 tubular adenocarcinoma, 25 poorly differentiated adenocarcinoma, 6 mucinous adenocarcinoma, 10 signer-ring cell carcinoma, and 4 anaplastic carcinoma, and 32 patients had metastasis through lymph node. 46 healthy volunteers were studied simultaneously as control, including 17 females and 29 males ranging in age from 30 to 60 years. This study was approved by the ethical committee of the Affiliated People's Hospital of Jiangsu University, and written informed consent was obtained from all individuals.

### 2.2. Blood Samples

Peripheral blood samples were collected from healthy volunteers and patients. The collection tubes contained 0.2 mL sodium heparin. Peripheral blood mononuclear cells (PBMCs) were obtained by standard Ficoll-Hypaque density centrifugation (T lymphocytes occupy about 70%) and stored at −70°C for measurement of cytokines and transcription factors. 

Flow cytometer was used to assess frequencies of CD4^+^ or CD8^+^ T cells in PBMCs. In brief, PBMC samples were thawed and cultured for 24 h to allow cells to rest and reexpress cell surface receptors, and then CD3-PE-Cy5, CD4-PE, and CD8-FITC monoclonal antibodies (eBioscience, USA) were used to perform fluorescent antibody staining as our previous description [2]. The BD FACSCalibur system (Becton Dickinson FACSCalibur, USA) was used for acquisition and analysis of flow cytometry data. 

### 2.3. Design of Primers and Response Conditions

According to Genebank sequences, the genetic testing primers were designed by Premier 5.0 software and synthesized by Shanghai Sangon Biological Engineering Technology and Service Company. All sequences of primers are shown in Table 1.

### 2.4. RNA Extraction and cDNA Synthesis

Following the manufacturer's instructions, total RNA from PBMCs was extracted with Trizol (Invitrogen, USA). cDNA was synthesised with reverse transcription reagent kits (TOYOBO, Japan). All RNA samples were heated at 65°C for 10 min to denature the secondary structure with the template and cooled immediately on ice for 5 min. Total RNA (1 *μ*g) was reversely transcribed in a total volume of 20 *μ*L, containing oligo (dT) 1 *μ*L, dNTP (10 mM) 1 *μ*L, 5× RT buffer 4 *μ*L, ReverTra Ace (100 U/*μ*L) 1 *μ*L, RNase Inhibitor 1 *μ*L, DEPC free H_2_O added up to 20 *μ*L, and response conditions: 42°C 20 min; 99°C 5 min; 4°C 5 min. The cDNA was stored at −20°C.

### 2.5. Construction of Recombinant Plasmid Calibrator

PCR amplification was performed in the Thermo Hybaid System (Eppendorf, USA). The program consisted of an initial denaturation step for 5 min at 94°C followed by 30 cycles, with each cycle consisting of a 30 s denaturing step at 94°C, a 30 s annealing step at 56°C, and a 30 s extension step at 72°C. The reaction was completed by a final 5 min extension step at 72°C.

Purified *Hlx, T-bet*, *IFN*-*γ*, *Runx3*, and *β*-*actin* PCR fragments were transformed to PMD18-T vector (Invitrogen, USA) to establish recombinant plasmids PMD18-*T-bet*, *Hlx,* IFN-*γ*,* Runx3* and *β-actin*. All these recombinant plasmids were transformed into competent *E. coli* DH5*α* and transferred on a 1.5% agar Amp-resistant plate and then cultured at 37°C for 12 h–14 h. Positive clones were initially identified by sequence. Part of positive clones were further amplified and extracted, and accurately quantified with a nucleic acid-protein ultraviolet instrument. 10-fold serial dilution of the recombinant plasmid DNA were used as calibrator and stored at −20°C until use.

### 2.6. QRT-PCR for Detecting Objective Genes Expression

The expression of objective genes (*Hlx, T-bet*, *Runx3*, *IFN-*γ**) were detected by quantitative real-time polymerase chain reaction (QRT-PCR), all samples were calibrated by *β*-*actin*. All PCR reactions were performed using the Rotor-Gene 6000 System (Corbett Research, Australia) in a total volume of 25 *μ*L, containing 1 *μ*L cDNA, 0.5 *μ*L 1 : 1000 sybr1 (Takara, China), 0.5 *μ*L 10 *μ*M each primer, 10× PCR reaction buffer 2.5 *μ*L, dNTP Mix (10 mM each) 2.0 *μ*L, LA Taq DNA polymerase 0.3 *μ*L, and 17.7 *μ*L water. The concentration of* Hlx*, *T-bet*, *IFN-*γ**, *Runx3*, and *β-actin* transcripts in samples was calculated with the Corbett software according to corresponding standard curves. According to each standard curve, the expression levels of *Hlx, T-bet*, *IFN-*γ**, *Runx3*, and *β-actin* were obtained. The ratio of *Hlx, T-bet*, *IFN-*γ**, and *Runx3* mRNA expression levels were regarded as indicator for the levels of *β*-*actin*. All samples were measured in triplicate.

### 2.7. Immunohistochemical Analysis for Testing p53 Mutant in Gastric Carcinoma Sufferers

SP (streptavidin-peroxidase) method was used to detect the p53 mutant by commercially obtained mouse antihuman p53 kit (purchased from Beijing Zhongshan Biological Technology Limited Company, China) according to the manufacturer's instructions. Paraffin-embedded samples were cut into 5 *μ*m sections. The sections were microwaved in 10 mM citrate buffer (pH 6.0) for counterstained with hematoxylin. Negative controls were performed in all cases omitting primary antibody.

Evaluation of immunohistochemistry results: the cytoplasm and/or nucleus brown were regarded as p53 mutant positive. Under the light microscope, 5 fields were randomly selected and total number of 500 cells were counted. The positive cell percentage more than 25% indicated as positive staining and less than 25% were as negative.

### 2.8. Enzyme-Linked Immunosorbent Assay (ELISA) for Plasma IL-4 and IFN-*γ*

Plasma levels of IL-4 and IFN-*γ* were measured by ELISA, following the manufacturer's protocols (eBioscience, USA). All samples were measured in triplicate.

### 2.9. Statistical Analysis

All statistical analysis was performed using SPSS17.0 statistical analysis software. Data are expressed as the mean ± standard deviation (SD) in text and figures. Comparisons between paired or unpaired groups were performed using the appropriate Student's *t*-test. For nonparametric data, differences between two groups were analyzed by the Mann-Whitney U test. Spearman's correlation was used to test correlation between two continuous variables. *P* < 0.05 was considered to be statistically significant.

## 3. Results

### 3.1. The Proportions of Lymphocytes in the Peripheral Blood

The percentage of CD3^+^T cells was 71.9% in healthy volunteers and 68.2% in patients with gastric cancer, there was a significant difference between two groups (*P* < 0.05). The percentage of CD3^+^CD4^+^/CD3^+^CD8^+^ T cells was 43.5%/21.6% in healthy volunteers and 41.3%/22.1% in the patients, respectively; it showed that the percentage of CD3^+^CD8^+^ T cells was no significant difference between healthy control and gastric cancer (*P* > 0.05), but the percentage of CD3^+^CD4^+^ T cells was significantly decreased in patients. 

### 3.2. Electrophoresis Identification of PCR Amplification Fragment

**Figure 1:**
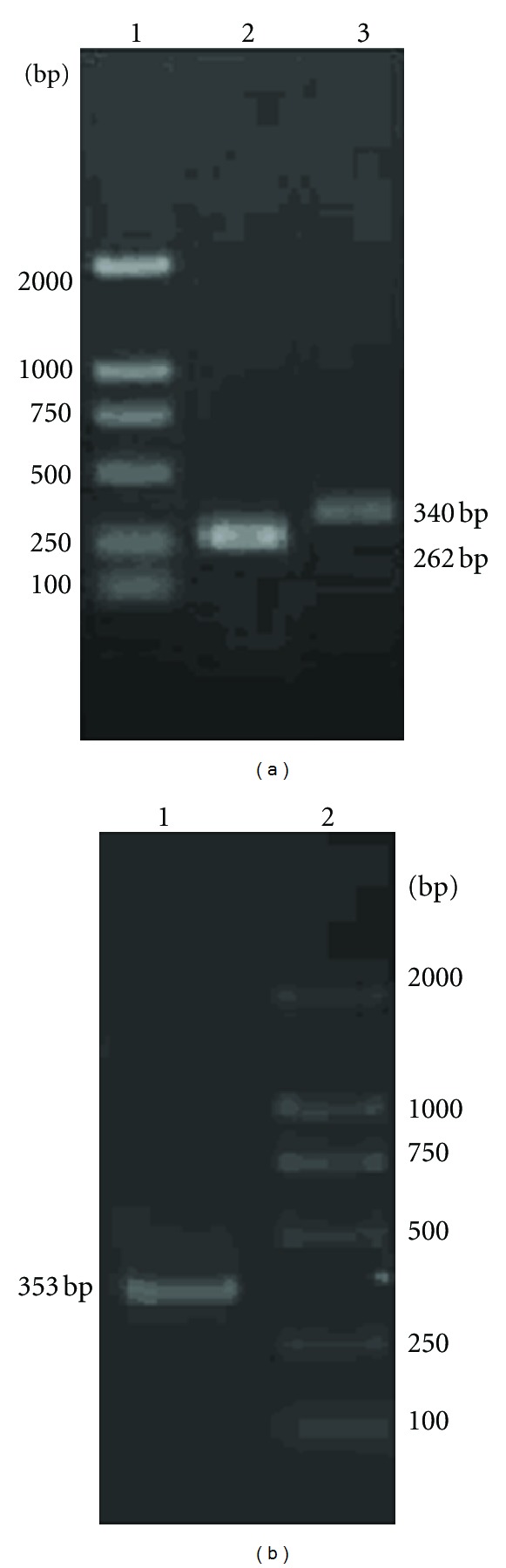
RT-PCR products of *Hlx *and* Runx3 *gene. (a) Lane 1, DNA marker DL2000, Lane 2, the PCR products of *β-actin* gene, and Lane 3, the PCR products of *Hlx*. (b) Lane 1, the PCR products of *Runx3*, Lane 2, DNA marker DL2000.

The length of the amplified target fragment of *Hlx*, *T-bet*,* Runx3, GATA3*, and **β*-actin* was 340, 339, 353, 309, and 262 bp, respectively, and it was consistent with the expected ones. The RT-PCR products of *Hlx* and *Runx3 *genes were shown in Figure 1. Identification of positive clone recombinant plasmids was validated by sequence. These objective gene sequences were in accordance with the ones provided by the National Center of Bioinformatics Institute (NCBI) (detailed data not shown). 

### 3.3. The Levels of **Hlx **, *T-bet*, *IFN-*γ**, and *Runx3* mRNA in Individual Patient with Gastric Cancer

**Figure 2:**
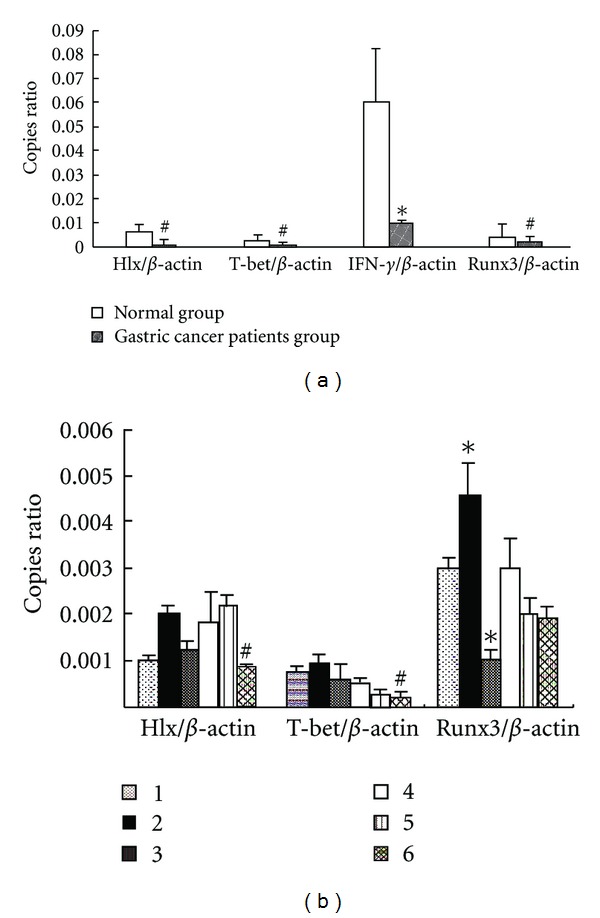
The expression levels of *Hlx*, *T-bet*, *Runx3,* and *IFN-*γ** mRNA in PBMC from gastric carcinoma sufferers and control. The values were presented as relative expression ratio (copies ratio) which means expression for the target transcript versus reference transcript (*β*-*actin*, a house keeping gene). The copies ratio showed that the *Hlx*, *T-bet*, *Runx3*, and *IFN-*γ** expression were decreased in patients with gastric cancer, compared with healthy control (a). The expression levels of the three kinds of transcription factors (*Hlx*, *T-bet*, and *Runx3*) in the different groups of patients (b). 1: papillary adenocarcinoma (*n* = 12); 2: tubular adenocarcinoma (*n* = 33); 3: poorly differentiated adenocarcinoma (*n* = 25); 4: mucinous adenocarcinoma (*n* = 6); 5: signer-ring cell carcinoma (*n* = 10); 6: anaplastic carcinoma (*n* = 4). *P < 0.01 compared with other groups; ^#^ *P* < 0.05 compared with papillary adenocarcinoma and tubular adenocarcinoma groups.

The expression levels of *Hlx*, *T-bet*, *IFN-*γ**,* and Runx3* mRNA from 90 patients with gastric cancer and 46 healthy controls were measured by QRT-PCR. As shown in Figure 2(a), the mRNAs of *Hlx*, *T-bet, Runx3*, and *IFN-*γ** were significantly decreased in peripheral blood mononuclear cells from patients with gastric cancer compared with healthy controls (*P* < 0.05, *P* < 0.05, *P* < 0.01, and *P* < 0.05). 

We have compared detection index (including the mRNA of *T-bet*, *Hlx*, and *Runx3*) with histological classification and analyzed the statistical significance that arose from different histological types. The decreased expression of *Hlx*, *T-bet*, and *Runx3* were found in each histological type of gastric cancer. In tubular adenocarcinoma group, *Hlx*, *T-bet*, and *Runx3* expression levels were higher than those of other patient groups, although these indicators were lower than that in healthy control. The level of *Hlx* and *T-bet* mRNA was the lowest in patients with anaplastic carcinoma, it was statistically significant compared with papillary adenocarcinoma and tubular adenocarcinoma groups (*P* < 0.05). The lowest and highest *Runx3* mRNA expression occurred in poorly differentiated adenocarcinoma group and tubular adenocarcinoma group, respectively (Figure 2(b)). 

### 3.4. The Correlation between the mRNA Levels of *Hlx*, *T-bet*, and *GATA3*

**Figure 3:**
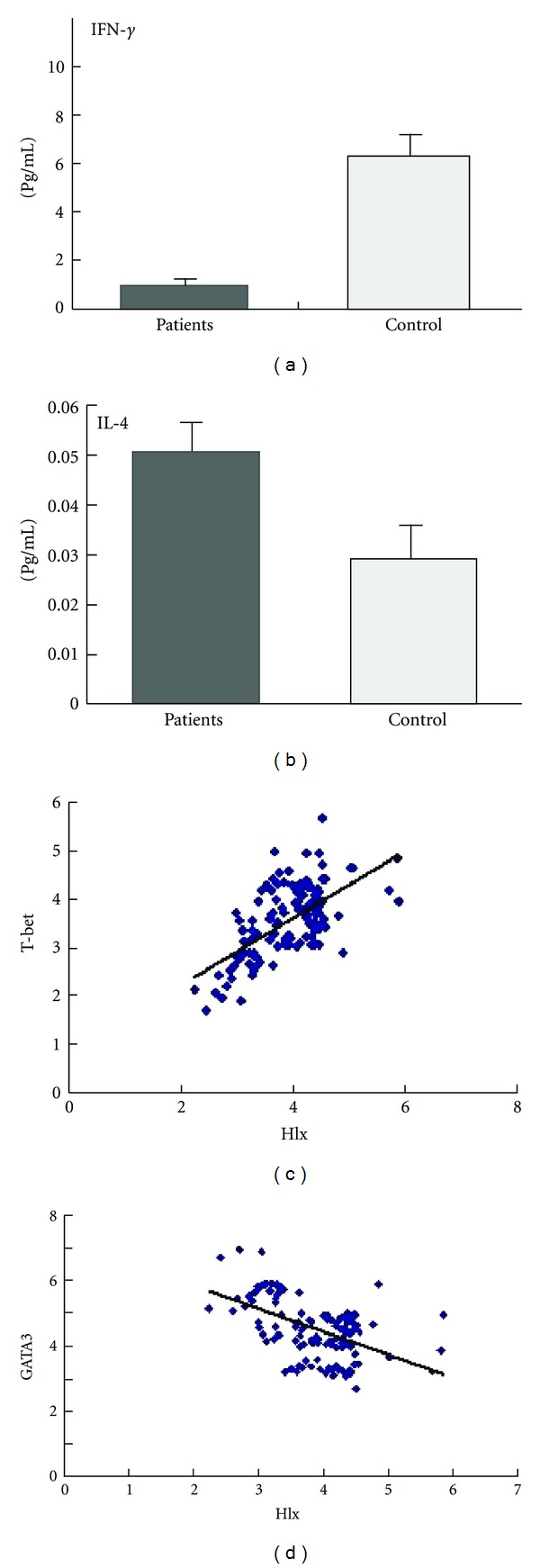
The levels of IFN-*γ* and IL-4 in serum and the correlation between expression levels of *Hlx* and *T-bet/GATA3* mRNA from gastric carcinoma sufferers. In serum of patients, the level of IFN-*γ* was obviously decreased (*P* < 0.01), but the level of IL-4 was enhanced (*P* < 0.01), compared with healthy control (a) and (b). The vertical coordinate indicates the* T-bet/*β*-actine* ratio (c) or *GATA3*/*β-actine* ratio (d), and horizontal coordinate indicates the *Hlx/*β*-actine* ratio. There was a significantly positive correlation between *Hlx *and *T-bet* (*r* = 0.635, *P* < 0.01), and a negative correlation between *Hlx* and *GATA3* (*r* = −0.523, *P* = 0.01).

We examined the correlation between the mRNA levels of *Hlx* and *T-bet* or *GATA3* in PBMCs of gastric cancer patients (90 cases). The results showed that the decreased level of *Hlx* mRNA was in line with *T-bet*, and the level of *GATA3* mRNA was increased following *Hlx* mRNA decreased (Figure 3). In addition, the level of plasma IFN-*γ* was decreased and the plasma IL-4 was enhanced in patients (Figure 3).

### 3.5. The Correlation between the mRNA Levels of *Runx3* and *T-bet* or *IFN-*γ**

**Figure 4:**
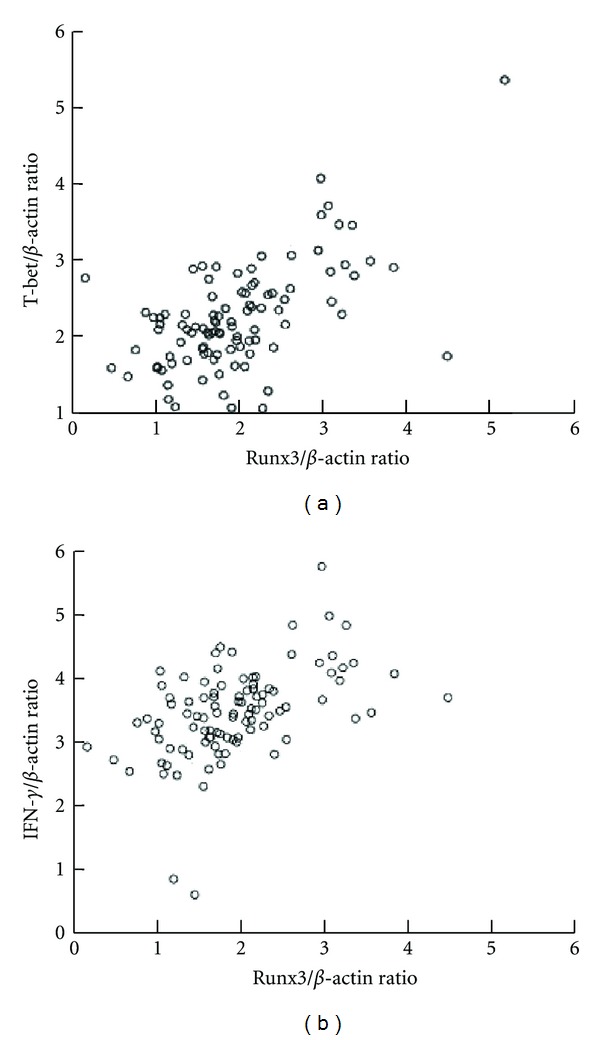
The correlation of *Runx3* and *T-bet* mRNA expression on PBMC from gastric carcinoma sufferers presented relative to control expression. (a) The correlation of *Runx3* and *T-bet* mRNA expression in gastric carcinoma patient, there was a positive correlation between *Runx3* and *T-bet* (*r* = 0.525, *P* < 0.01). (b) The correlation of *Runx3* and *IFN-*γ** mRNA expression in gastric carcinoma patient, there was a positive correlation between *Runx3* and *IFN-*γ** (*r* = 0.543, *P* < 0.01).

*Runx3* is a novel tumor suppressor in gastric carcinogenesis and an important factor for differentiation of chief cells in the normal gastric fundic mucosa. In addition, *Runx3* is a mechanism that causes the negative regulation of IL-4, along with previously reported repression by *T-bet*. In order to understand the relationship between the *Runx3* and *T-bet* expressions in gastric carcinoma sufferers, we analyzed the *Runx3 *mRNA levels in PBMCs of patients. Our data indicated that the expression levels of *Runx3* were significantly decreased and there was a positive correlation between *Runx3* and *T-bet* or *IFN-*γ** (Figure 4).

### 3.6. The Relationship between p53 Mutant and *T-bet* or *GATA3* mRNA Levels in Gastric Carcinoma Sufferers

**Figure 5:**
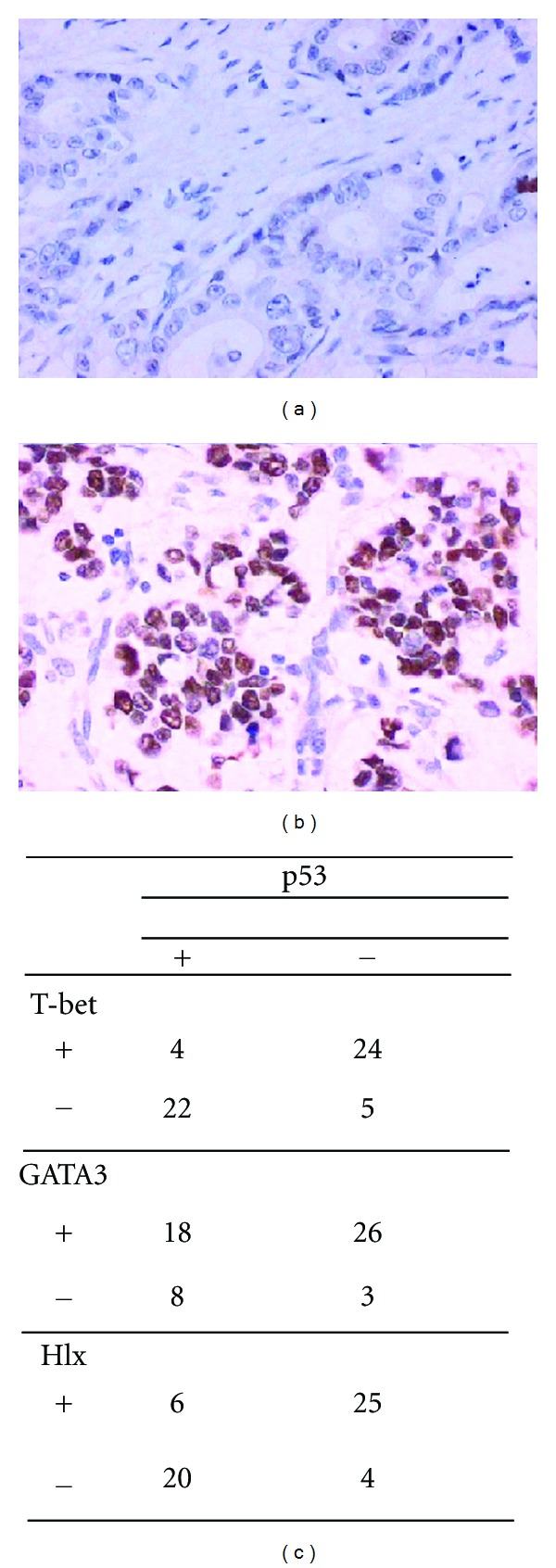
The p53 expression in patients with gastric carcinoma and its comparison with the levels of *T-bet*, *Hlx*, and *GATA3.* (a) and (b) showed the results of immunohistochemical analysis, the expression of p53 could be found in carcinoma tissue in some patients (a), but some of them showed negative expression (b). (c) summarized the comparison between p53 expressed in gastric cancer tissue and *T-bet*, *Hlx*, or *GATA3 *mRNA levels in PBMC from the same patients.

The histological sections from 55 cases of patients were used to analyze the expression of p53 mutant, and the *T-bet* and *GATA3* mRNA levels detected from the same patient were used to make a comparative study. As shown in Table 2, the expression level of intact p53 and mutant p53 was 70% and 47%, respectively. Further analysis displayed that in total p53 mutant expression p53 mutant positive and *T-bet* negative patients were 22 cases (40%), it included 10 cases of patients with widespread metastases. However, there were 24 cases with p53 mutant negative plus *T-bet* positive (44%). In p53 mutant positive patients, only 4 were *T-bet* positive and 5 were *Hlx* positive. The Spearman rank test showed the *T-bet/Hlx* and p53 mutant expression levels were negatively correlated (*r* = −0.6728, *P* < 0.01); *GATA3* and p53 mutant expression levels were positively correlated (*r* = 0.736, *P* < 0.01) (Figure 5).

## 4. Discussion 

The precise mechanism of the development of gastric carcinoma is not very clear yet. But it is accepted that the reason was synergistic effect of extrinsic and intrinsic factors, and the switch of immune state as one of the explanation of the development of gastric carcinoma is paid great attention to [13–16]. In normal physiological state, the specific factors secreted by Th1 cells are IL-2 and IFN-*γ*, and the factors of Th2 are IL-4 and IL-10. Th1 plays an important role in enhancing response in cell-mediated immunity. In addition, the effect of Th2 cells is to mediate humoral immunity. The cross regulation and interrestraint between these two cells is very essential to maintain the balance of immune system. The co-precursor cell is Th0 cells, when Th0 cells are stimulated by antigen, proportional differentiation will occur. *T-bet* and *GATA3* are two transcriptional factors, respectively, charging the differentiation of Th0 cells to Th1 or Th2 cells. When disorder occurs in immunomediation, corresponding Th1 or Th2 drifting disease will be developed because of the Th0 cells differentiation disequilibrium. It is reported that Th2 drifting is related to immune escape of tumor [17, 18]. *Hlx* as a positive regulator of IFN-*γ* production in CD4+ T lymphocytes and *T-bet* target, it induces the differentiation of Th1 and blocks Th2 commitment together with *T-bet *[19]. In addition, the expression of *Hlx* strongly reduces the capacity of the cells to form vessels, it is prompted that *Hlx* may relate to tumor metastasis [20].* Runx3* has been reported to be a candidate tumor suppressor gene in gastric cancer and also a regulator of Th1 cells. *p53 * gene is a kind of tumor suppressor gene; the production of *p53* gene expression is called *p53* protein; its activity stops the formation of tumors [21, 22]. However, the immunohistochemistry detected *p53* protein is mainly the mutant, which is closely related to the tumor cell proliferation, invasion, and metastasis. 

We used to judge the balance of Th1/Th2 via the level of cytokine, but recent research proved that the analysis of some transcription factors can tell more information [23]. In the present study, we tried to explain the drifting of Th cells by analyzing the expression of transcription factors and associated cytokines in gastric carcinoma, and analyzed the relationship between them. Our results showed that *T-bet*,* Hlx, Runx3*, and *IFN-*γ** expression in transcription and protein levels were significantly lower in gastric carcinoma group than that in control group, but the results of *GATA3* and *IL-4* were opposite, which could demonstrate that the decreasing cell-mediated immune ability from gastric cancer state and a typical Th2 cells drifting had happened in gastric carcinoma. We also compared the detection index including the mRNA of *T-bet* and *Hlx* with histological classification; the decreased expression of the two kinds of transcription factors were found in each histological type of gastric cancer, and it suggested that regardless of histological type of gastric cancer, there were Th1/Th2 imbalance. In addition, our observation that the lower expression of* T-bet* and *Hlx* were more marked in anaplastic carcinoma than in tubular adenocarcinoma corroborated that the Th1/Th2 cells imbalance was most pronounced in malignant cancer. The correlation analysis indicated that there was a significantly positive correlation between *Hlx *and *T-bet*, and a negative correlation between *Hlx* and *GATA3*. Our data also showed that the expression rates of *Runx3* in patients with gastric cancer (especially poorly differentiated adenocarcinoma) were obviously lower than that in control groups and there was a positive correlation between the expression of *Runx3* and *T-bet* or *IFN-*γ** in patient. The patients expressed p53 mutant accompanyed with lower expression rate of *T-bet* and *Hlx*, the negative correlation existed between p53 mutant and *T-bet or Hlx *in gastric cancers. 

Metastasis is one of the main causes of death in patients with gastric cancer. Our research found that some transcription factors played a key role in metastasis related gene regulation. Some scholars reported that the cancer cell proliferation rate was greatly enhanced in *T-bet* knockout mice. p53 mutant was closely related to tumor cell proliferation, invasion, and metastasis. This study also found that the positive rate of p53 mutants was significantly higher in the tissue of gastric carcinoma with lymph nodes metastasis than those without lymph node metastasis. *T-bet* and p53 mutant frequency analysis discovered that *T-bet*/*Hlx* and p53 mutant expression levels were negatively correlated, and *GATA3* and p53 mutant expression levels were positively correlated. It also indicated that the Th2 cell polarization was raised in gastric carcinoma and related to the expression of p53 mutant which associated with tumor cell proliferation, invasion, and metastasis.

It is well known that the decreased Th1 cell-mediated immunity is related to immune escape of tumor. In this experiment, we supposed that the predominant expression of Th2 type cytokines was related to lower expression of *T-bet* or higher expression of *GATA3* in PBMC from patients with gastric cancer. The induction of *T-bet* might be useful in two main respects, one was upregulating Th1 cell response, enhancing cell-mediated immunity in the person suffered form cancer, another was inevitably strengthening the effect on killing tumor cells by increasing IFN-*γ* production. In addition, enhanced p53 mutant frequency might be due to Th1 cell downregulation and associated with invasion and metastasis of gastric carcinoma. All the above results showed that the *T-bet* associated biological events were closely related to the expression levels of *Hlx* and *Runx3* and the development of gastric cancer. *Hlx* is a positive regulator of *T-bet* and *IFN-*γ** and its expression in patients with gastric carcinoma was probably influenced by the local environment, including cytokine patterns and tumor antigen, and further studies would be necessary to clarify these points.

## Figures and Tables

**Table 1:** The detection primer sequence and amplified range.

Gene	Primer and probe	Length (bp)
*T-bet *	For: 5′-GTTCCCATTCCTGTCSTTTACT-3′	339
	Rev: 5′-TCTCCGTCGTTCACCTCAA-3′	

*GATA3 *	For: 5′-CTGTGGGCTGTACTACAAGCTTCA-3′	309
	Rev: 5′-ACCCATGGCGGTGACCATGC-3′	
*IFN*-*γ*	For: 5′-TATTCGGTAACTGACTTG-3′	378
	Rev: 5′-AATCACATAGCCTTGC-3′	
*Hlx *	For: 5′-GGCCAGTTCTTCGCATCTC-3′	340
	Rev: 5′-AGTGCCGCCACTTCATCC-3′	
*Runx3 *	For: GATGGCAGGCAATGACGA	353
	Rev: TGCTGAAGTGGCTTGTGGT	
*β*-*actin *	For: 5′- CACGAAACTACCTTCAACTCC-3′	262
	Rev: 5′- CATACTCCTGCTTGCTGATC-3′	

**Table 2:** The expression level of intact and mutant p53.

p53	Mutation		Total
	+	−
+	23	16	39
−	3	13	16

Total	26	29	55
